# Locally Advanced and Unresectable Cutaneous Squamous Cell Carcinoma: Outcomes of Concurrent Cetuximab and Radiotherapy

**DOI:** 10.1155/2014/284582

**Published:** 2014-07-21

**Authors:** Robert M. Samstein, Alan L. Ho, Nancy Y. Lee, Christopher A. Barker

**Affiliations:** ^1^Department of Radiation Oncology, Memorial Sloan Kettering Cancer Center, 1275 York Avenue, Box 22, New York, NY 10065, USA; ^2^Department of Medicine, Memorial Sloan Kettering Cancer Center, New York, NY 10065, USA

## Abstract

*Background.* Advanced age and immune dysfunction are risk factors for cutaneous squamous cell carcinoma (cSCC) and often render patients with locally-advanced disease medically inoperable or surgically unresectable, but potentially curable with radiotherapy. Concurrent chemotherapy and radiotherapy may not be well tolerated in this population, but another systemic therapy may improve disease control. *Objective.* Determine the tolerance and efficacy of concurrent cetuximab and radiotherapy (CRT) for patients with locally advanced and unresectable cSCC. *Methods.* Retrospective analysis of 12 patients treated with CRT for locally advanced and unresectable cSCC. *Results.* Patients were elderly and 75% had moderate-to-severe comorbidities, while 42% had immune dysfunction. Grades 3-4 adverse events were noted in 83% of patients; 67% required hospital admission for adverse events. Complete and partial response was noted in 36% and 27% (response rate, 64%). Stable and progressive disease was noted in 3 and 1 patients, respectively (disease control rate, 91%). Median progression-free and overall survival were 6.4 and 8.0 months, respectively. *Limitations.* Retrospective small-cohort, single-institution analysis. *Conclusion.* Patients selected for CRT were elderly, with comorbidities and immune dysfunction, but treatment responses were observed. Patients selected for this treatment approach have a poor prognosis with limited capacity for therapy; more effective treatment is needed.

## 1. Introduction

Cutaneous squamous cell carcinoma (cSCC) is one of the most common cancers in the United States with an increasing incidence over the past few decades. The disease often presents at an early stage and is controlled with surgical, radiation, topical, or photodynamic therapy. Advanced age and immune dysfunction are risk factors for cSCC and render some patients medically unfit for surgery at diagnosis or recurrence. Moreover, some patients present with extensive local invasion or metastasis, rendering the cSCC surgically unresectable. Patients with locally advanced cSCC that are medically inoperable or surgically unresectable have a poor prognosis but can be cured with radiotherapy [1, 2].

Improving the outcome of radiotherapy through the use of concurrent systemic therapy has been demonstrated in several locally advanced cancer-treatment paradigms. Platinum (e.g., cisplatin, carboplatin) and halogenated pyrimidine (e.g., 5-fluorouracil) chemotherapies are frequently used in conjunction with radiotherapy to improve treatment efficacy but may not be well tolerated by patients of advanced age, or those who are immunosuppressed or harbor significant comorbidities [3]. For this particular patient population, a systemic therapy to combine with radiotherapy that is effective and well tolerated is needed.

Cetuximab (Erbitux, Genentech) is a monoclonal chimeric IgG1 antibody that binds and blocks the epidermal growth factor receptor (EGFR). EGFR, a transmembrane tyrosine kinase, has been shown to be upregulated in a variety of squamous cell carcinomas and its downstream antiapoptotic signaling cascade has been well studied [4]. In cSCC, series have reported EGFR overexpression in 43–100% of patients studied [5–8], and overexpression appears to be more common in patients with metastasizing cSCC [9]. Reports from small clinical trials have indicated that cetuximab has activity in metastatic or unresectable cSCC, either alone or in combination with other therapies [10, 11].

Cetuximab has been approved by the Food and Drug Administration for use in combination with radiotherapy for mucosal squamous cell carcinoma of the head and neck based on a large randomized trial demonstrating improved survival compared with radiotherapy alone [12, 13]. Cetuximab is thought to function as a radiosensitizer contributing to a synergistic effect when it is combined with radiotherapy [14]. The combination of cetuximab and radiotherapy (CRT) has also been tested in several other EGFR-expressing squamous cell carcinomas including lung, anal, esophageal, and uterine cervix squamous cell carcinoma [15–18]. There is little data available on the safety and effectiveness of CRT in patients with advanced cSCC. We thus sought to retrospectively study the toxicity and efficacy of combination CRT in patients with advanced cSCC treated at our institution.

## 2. Methods

### 2.1. Patients

Review of medical records was conducted with permission of the institutional review board (WA0552-11). Patients with cSCC that were selected for treatment with CRT were identified. Only patients that underwent concurrent treatment with both modalities were included in the study.

Patient demographics, comorbidities, and details of cSCC diagnosis and stage at the time of CRT were recorded. Comorbidities were classified according to the Adult Comorbidity Evaluation-27 (ACE-27). This system identifies 27 common medical ailments among 12 organ systems or disease processes and provides criteria to grade the comorbidity on a scale of 0–3 (0, ailment not present; 1, mild decompensation; 2, moderate decompensation; 3, severe decompensation) [19]. Staging was performed according to the cutaneous squamous cell carcinoma system in the American Joint Committee on Cancer Staging Manual, version 7 [20].

### 2.2. Treatment and Adverse Events

Details of prior treatment including surgery, radiotherapy, and systemic therapy were reviewed and recorded. Common Terminology Criteria for Adverse Events version 4.0 (CTCAE v4.0) was used to assess, characterize, and grade adverse events observed during both cetuximab and radiotherapy [21].

### 2.3. Treatment Response

Treatment response within and outside the irradiated volume was assessed at the first posttreatment clinical and radiographic evaluations, 4–12 weeks after the completion of therapy. Formal imaging response assessment was not possible in some patients who were followed clinically (without imaging) after therapy and because of heterogeneous follow-up imaging. Operational definitions based on the Response Evaluation Criteria in Solid Tumors (RECIST) and PET Response Criteria in Solid Tumors (PERCIST) were used and included complete response (CR, disappearance of lesion), partial response (PR, 30% decrease in longest dimension of lesion), stable disease (SD, no evidence of response or progression), and progressive disease (PD, 20% increase in longest dimension of the lesion). Overall and cSCC-specific survival were recorded.

### 2.4. Statistical Analysis

Kaplan-Meier curves were generated and used to estimate survival rates (with asymmetric 95% confidence intervals) and median survival times and to compare between groups of patients. Statistical analysis was conducted using Graphpad Prism v6.0c.

## 3. Results

### 3.1. Patients

Twelve patients were selected for treatment with concurrent cetuximab and radiotherapy for locally advanced or unresectable cSCC between 2007 and 2013. Three patients were excluded from analysis: one received induction systemic therapy with cetuximab, carboplatin, and paclitaxel, followed by radiotherapy alone; two patients received palliative cetuximab for distant metastases and received a brief course of palliative radiotherapy directed at a site of distant metastasis. As detailed in Table 1, most patients were elderly (median age, 78 years; range, 47–90), all were white, and all but one was male. Median Karnofsky performance score was 80 (range, 50–90). Most patients had moderate (42%) or severe (33%) comorbidities. Almost half (42%) of patients had identifiable immune dysfunction (chronic lymphocytic leukemia in 4, solid organ transplant in 1, and acquired immunodeficiency syndrome in 1).

The stage and presentation of cSCC is presented in Table 2. Most patients (75%) received CRT for recurrent cSCC. All but two patients with known primary tumors (82%) underwent excision; 4 of 9 patients had nodal recurrence after prior lymphadenectomy. No patients received prior chemotherapy for cSCC. All but one patient (who was given adjuvant CRT after surgical resection) had gross disease present at the start of treatment. All patients had locally advanced cSCC (T4 tumors) or regional nodal metastases. Two patients had distant metastases at the start of CRT.

### 3.2. Treatment and Adverse Events

**Figure 1:**
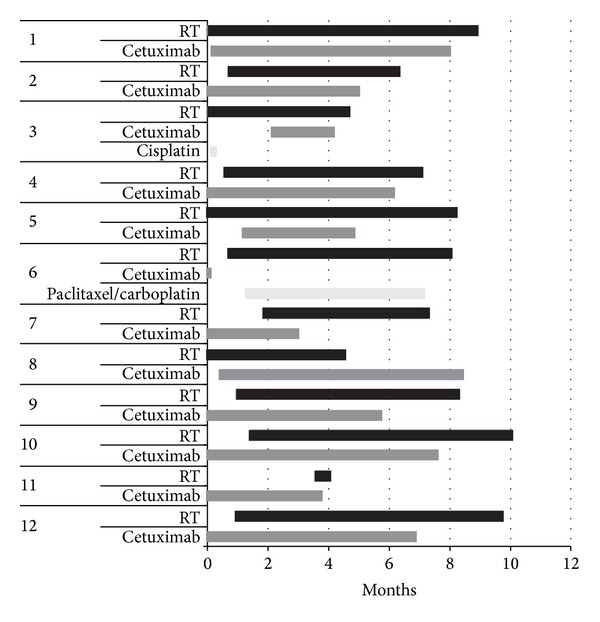
Relationship of cetuximab and radiotherapy. The duration of treatment and relationship in time for radiotherapy, cetuximab, and other systemic therapies is presented.

All patients were treated with static-field intensity modulated radiation therapy using dynamic multileaf collimation. Treatment was delivered by a linear accelerator producing 6 MV photons or 6–9 MeV electrons, depending on the treatment target. One patient (number 3) was initially selected for concurrent cisplatin and radiotherapy but did not tolerate this and was switched to CRT. Conversely, one patient (number 6) was initially selected for CRT but did not tolerate this and was switched to carboplatin and paclitaxel concurrent with radiotherapy. The duration and relationship between cetuximab administration and radiotherapy are plotted in Figure 1. Radiation doses ranged between 12 and 80 Gy with a median dose of 60 Gy in 30 fractions (range, 3–38). Patients received cetuximab at 400 mg/m^2^, followed by weekly treatment at 250 mg/m^2^ through the end of radiotherapy, if they tolerated this treatment approach. Median cetuximab dose was 1525 mg/m^2^ (range, 400–2400). Treatment was delayed in 5 patients due to adverse events, and 2 patients had radiotherapy terminated early due to progression of disease. Eight patients were hospitalized during or soon after treatment. The frequency of grades 2–4 adverse events is shown in Table 3; no patient developed a grade 5 adverse event although 83% of patients experienced a grade 3 or higher event. The most common adverse events observed included fatigue, acneiform rash, radiation dermatitis, and infection.

### 3.3. Treatment Response

The best clinical response in the irradiated volume among the 11 patients treated for gross disease (i.e., not adjuvantly) was CR in 4 patients (36%) and PR in 3 (27%), for an overall response rate of 64% (95% confidence interval, 35–92%). Median time to progression within the irradiated volume for patients achieving CR and PR was 9.9 and 6.4 months, respectively. Within the irradiated volume, SD was noted in 3 patients and PD in another patient, for a disease-control rate (DCR) of 91% (95% confidence interval, 74–100%). Both of the patients with distant metastases at the start of CRT had PD outside of the irradiated volume. Among patients without distant metastases at the start of CRT, PD occurred within the irradiated volume in 5 and outside the irradiated volume in 4. The patient treated with adjuvant CRT died 4.8 months after treatment of noncancer-related causes with no evidence of recurrent cSCC.

**Figure 2:**
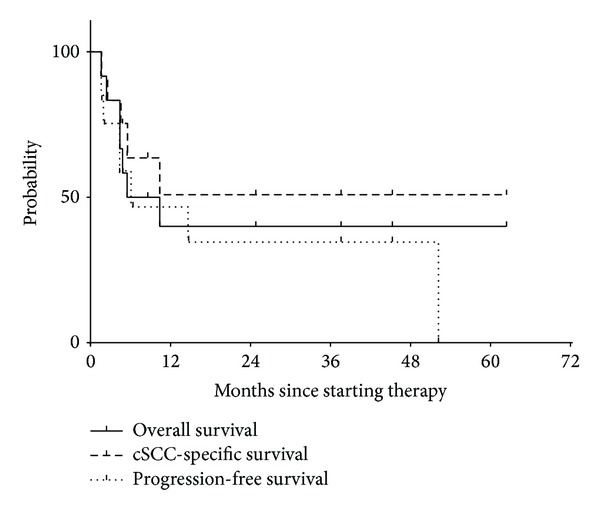
Overall, disease-specific, and progression-free survival. Median progression-free and overall survival were 6.4 and 8.0 months, respectively. Median cutaneous squamous cell carcinoma- (cSCC-) specific survival was not reached. cSCC-specific and overall survival were 51% (95% confidence interval, 26–85%) and 40% (95% confidence interval, 14–66%) at 2 years.

As noted in Figure 2, at the time of analysis, 7 of 12 (58%) patients studied had died. Five of 7 (71%) died of cSCC. Median follow-up for the entire cohort was 7.0 months but was 37.6 months for the 5 surviving patients. Median progression-free and overall survival were 6.4 and 7.95 months, respectively. Median cSCC-specific survival was not reached. cSCC-specific and overall survival were 51% (95% confidence interval, 26–85%) and 40% (95% confidence interval, 14–66%) at 2 years. Median progression-free and overall survival were 2.1 and 3.6 months in patients with distant metastases at the start of CRT compared with 14.7 and 10.4 without distant metastases, respectively. Median overall survival of patients with immune dysfunction was 4.4 months, while it was not reached among patients without immune dysfunction. Likewise, median survival of patients with moderate and severe comorbidities was 5.5 and 3.4 months, respectively, while it was not reached in patients with mild comorbidities.

## 4. Discussion

This study was designed to assess the efficacy and safety of the combination of cetuximab and radiotherapy in patients with advanced cSCC. We found that this treatment strategy yielded a response in 64% of patients, although disease progression after response was common and survival was limited. We found that patients selected for this treatment strategy were often elderly, with comorbidities and immune dysfunction. Despite the development of moderately severe adverse events, many patients required hospitalization in the period of time surrounding treatment. These results suggest that the patients we have selected for this treatment approach have a poor prognosis and limited capacity for therapy.

A multicenter phase II study of cetuximab monotherapy for unresectable cSCC was published in 2011. Thirty-six patients were accrued from 2005 to 2008, all of whom had performance status ≥2 and no immune dysfunction. Median age of the group was 79 years (range, 32–95). Unresectable cSCC was present at the site of the primary tumor, regional lymph nodes, and distant metastasis in 47, 44, and 8% of the group, respectively. Six weeks after receiving cetuximab (400 mg/m^2^, then 250 mg/m^2^ weekly), 3% of patients had a CR, 8% of patients had a PR, and 58% had SD, for a DCR of 69% (95% confidence interval, 52–84%). The best overall response rates were CR in 6%, PR in 22% (response rate, 28%; 95% confidence interval, 14–45%), and SD in 42% (DCR, 69%; 95% confidence interval, 52–84%). Median progression-free survival was 4.1 months (95% CI 1.7–5 months). Twenty-three patients (64%) experienced a serious adverse event (grades 3-4). Acneiform rash (but not genetic polymorphisms) was associated with favorable progression-free survival [10].

A single-center phase II study of cetuximab for unresectable cSCC was recently published. Among 20 patients accrued between 2009 and 2011, 5 were selected for treatment with radiotherapy (60–70 Gy) in conjunction with cetuximab (400 mg/m^2^, followed by 250 mg/m^2^ weekly for 3 weeks). One of the 5 patients receiving CRT was immunosuppressed, and the median age of this subgroup was 77 years. After 2 cycles of therapy (8 weeks) using RECIST criteria the authors observed no patient to have a CR, 4 of 5 (80%) a PR, and 1 of 5 (20%) SD, for a DCR of 100%. Median progression-free survival was 5 months. Four (80%) patients experienced a serious adverse event (grade 3-4). Patients selected to receive radiotherapy appeared to have a higher response rate (80%) than patients selected to receive carboplatin with cetuximab (response rate, 44%) or cetuximab alone (response rate, 33%) [11].

Between 2010 and the present, we have identified 10 patients with cSCC treated with CRT and reported in the medical literature as case reports or small case series.  Table 4 provides details of 8 of these patients, in addition to the 5 patients treated with CRT from the phase II trial noted above. The median age of patients is 77 years (range, 61–85). Immune dysfunction was present in at least 1 patient. All had recurrent, unresectable cSCC. Most patients received cetuximab (400 mg/m^2^, then 250 mg/m^2^ weekly) for a median of 7 weeks and median radiation dose was 60 Gy. Six of eight (75%) patients from case reports or series were reported to have a complete response, while 2 of 8 (25%) were reported to have a partial response [22–26]. One patient treated for unresectable cSCC with CRT was described in another case series, but details were not reported [27]. Yet another case series reported a 62-year-old man treated with adjuvant CRT after resection of a locally advanced cSCC, with no evidence of recurrence 2 years after treatment [28].

The results of CRT described above are generally consistent with those in the present study. The studies have all reported groups of elderly patients, with a median age in the late eighth decade. Immune dysfunction was less common in the previously reported studies (0–20% of patients), compared with the present analysis (46.1%). Importantly, the prior studies of CRT did not report the presence of comorbidities, which were found to be moderate or severe in the majority (76%) of patients studied. Disease stage (and, specifically, the presence of distant metastasis) varied across the studies and is likely to be associated with the outcome of treatment. For these reasons, comparing the present results to prior studies is challenging. Nevertheless, the observed response rate of 64% is similar to the subset of patients in the recently published phase II study (80%).

Our study has several potential limitations. First, the number of patients studied was small (*n* = 12). However, to our knowledge, the present report is the largest single-institution experience of CRT of cSCC and nearly doubles the number of patients reported after treatment for cSCC with CRT in the medical literature. Second, the treatment approach varied among patients studied. This is a function of the extended time period during which this study took place, as well as the nuances of the specific patient and disease characteristics confronted by clinicians at our center. Nevertheless, cetuximab was given in a consistent fashion (400 mg/m^2^, followed by 250 mg/m^2^ weekly), and radiotherapy was generally given to curative doses in conventional fractionation (60–70 Gy in 30–35 fractions) using standard static-field intensity-modulated techniques. Third, the methods for assessing response were not standardized. However, all patients underwent clinical evaluation and imaging within a relatively limited window of time after the completion of treatment, consistent with the prospective studies on this subject. Finally, the retrospective nature of this study with inherent selection biases and lack of a control group limits the strength of the conclusions that can be made. This study should therefore be used to generate hypotheses for future testing.

## 5. Conclusion

Our study was designed to assess the safety and efficacy of CRT for patients with locally advanced or metastatic unresectable cSCC. We found that the treatment was delivered to a group of elderly patients with moderate-to-severe comorbidities who often harbored immune dysfunction. Nevertheless, the majority of patients exhibited response to treatment. However, progression of disease typically followed soon thereafter. Progression-free and overall survival were limited, probably as a cumulative result of advanced age, comorbidities, immune dysfunction, and advanced cancer. Additional studies are needed to further improve outcomes by reducing the morbidity and increasing the efficacy of treatment.

## Figures and Tables

**Table 1:** Patient characteristics.

Patient	Age	Sex	Race	Overall comorbidity severity	Moderate and severe comorbidities	KPS	Immune dysfunction
1	83	M	W	Mild		80	
2	82	F	W	Mild		80	CLL
3	70	M	W	Moderate	Respiratory	80	
4	59	M	W	Moderate	Cardiovascular (congestive heart failure, arrhythmia)	70	Heart transplant
5	78	M	W	Moderate	Obesity	80	
6	85	M	W	Moderate	Cardiovascular (arrhythmia)	80	
7	47	M	W	Severe	Immunologic (AIDS)	70	AIDS
8	78	M	W	Moderate	Malignancy (leukemia)	70	CLL
9	75	M	W	Severe	Endocrine (diabetes), respiratory, malignancy (leukemia)	90	CLL
10	77	M	W	Mild		80	
11	90	M	W	Severe	Malignancy (solid tumor)	50	
12	86	M	W	Severe	Malignancy (solid tumor)	60	

Demographic, comorbidity, and immune system dysfunction for each of the patients (*n* = 12) studied.

KPS: Karnofsky performance status, M: male, F: female, W: white, CLL: chronic lymphocytic leukemia, and AIDS: acquired immunodeficiency syndrome.

**Table 2:** Disease characteristics and treatment response.

Patient	Stage∗	Recurrent	Gross disease	Posttreatment response within the irradiated volume	Months until PD (in or out of irradiated volume)
1	T4N2bM0	No	Yes	PR	6.4 (in)
2	T0N2bM0	Yes	Yes	CR	14.7 (in), 16.3 (out)
3	T0N2bM0R0	Yes	No	N/A	(Died with NED)
4	T4N0M0	Yes	Yes	SD	(Died without PD)
5	T0N1M0	Yes	Yes	CR	(Alive with NED)
6	T4N0M0	Yes	Yes	SD	(Alive with NED)
7	T2N2bM0	No	Yes	PD (during treatment)	1.7 (in and out)
8	T0N2bM1	Yes	Yes	CR	2.1 (out), 5.0 (in)
9	T2N2bM0	Yes	Yes	CR	4.4 (out)
10	T4N0M0	Yes	Yes	SD	52.2 (in)
11	T0N3M1	Yes	Yes	PR	1.6 (out)
12	T4N0M0	No	Yes	PR	4.4 (out), 4.7 (in)

Disease status at the start of therapy and investigator assessed response 4–12 weeks after therapy are presented.

*All patients were staged clinically, except patient 3, who was staged pathologically.

PR: partial response, CR: complete response, SD: stable disease, PD: progressive disease, NED: no evidence of disease, IV: irradiated volume, and N/A: not applicable (because patient received adjuvant therapy [no measureable disease for response assessment]).

**Table 3:** Grades 2–4 adverse events occurring during cetuximab and radiotherapy classified according to the Common Terminology Criteria for Adverse Events, version 4.0; no grade 5 adverse events were observed.

Adverse event	Grade 2		Grade 3		Grade 4	
	*N*	(%)	*N*	(%)	*N*	(%)
Infusion reaction	1	(8)	0	(0)	0	(0)
Acneiform rash	5	(42)	0	(0)	0	(0)
Radiation dermatitis	5	(42)	3	(25)	0	(0)
Mucositis	3	(25)	2	(17)	0	(0)
Pneumonitis	0	(0)	1	(8)	1	(8)
Anemia	1	(8)	1	(8)	0	(0)
Thrombocytopenia	1	(8)	0	(0)	0	(0)
Neutropenia	0	(0)	1	(8)	0	(0)
Fatigue	7	(58)	1	(8)	0	(0)
Weight loss	2	(17)	0	(0)	0	(0)
Xerostomia	2	(17)	1	(8)	0	(0)
Dysphagia	3	(25)	1	(8)	0	(0)
Infection	2	(17)	3	(25)	0	(0)

**Table 4:** Reported cases of cSCC treated with CRT.

Study design	First author	Publication year	Case number	Age	Sex	Radiotherapy dose (Gy)	Duration of therapy (weeks)	Response	Disease-free survival (months)
Case report	Kanakamedala MR	2010	—	78	M	n/r	8	CR	5
Case report	Goppner D	2010	—	85	F	45	n/r (<12)	CR	14
Case report	Wollina U	2011	—	77	M	60	6	CR	3
Case series	Giacchero D	2011	2	67	M	50	7	CR	3
Case series	Giacchero D	2011	3	72	M	60	7	CR	5
Case series	Giacchero D	2011	4	79	M	70	18	CR	21
Case series	Giacchero D	2011	8	78	M	70	7	PR	n/r
Case series	Alter M	2013	2	61	M	24	5	PR	5
Phase II trial	Preneau S	2014	8	62	M	60–70	n/r	PR	8
Phase II trial	Preneau S	2014	11	63	M	60–70	n/r	PR	8
Phase II trial	Preneau S	2014	16	83	F	60–70	n/r	PR	4
Phase II trial	Preneau S	2014	17	86	F	60–70	n/r	PR	5
Phase II trial	Preneau S	2014	20	77	M	60–70	n/r	SD	5

M: male, F: female, n/r: not reported, PR: partial response, CR: complete response, SD: stable disease, and PD: progressive disease.

## Abbreviations

ACE-27: Adult Comorbidity Evaluation-27
CI: Confidence interval
CR: Complete response
CRT: Concurrent cetuximab and radiotherapy
cSCC: Cutaneous squamous cell carcinoma
CTCAE: Common Terminology Criteria for Adverse Events
DCR: Disease control rate
EGFR: Epidermal growth factor receptor
PD: Progressive disease
PERCIST: PET Response Criteria in Solid Tumors
PR: Partial response
RECIST: Response Evaluation Criteria in Solid Tumors
SD: Stable disease.
